# Absence during pregnancy in the Danish workforce: occupational, industrial, and temporal trends in a nationwide register-based cohort study

**DOI:** 10.5271/sjweh.4245

**Published:** 2025-11-01

**Authors:** Luise Mølenberg Begtrup, Esben Meulengracht Flachs, Regitze Sølling Wils, Ingrid Sivesind Mehlum, Jens Peter Ellekilde Bonde, Astrid Juhl Andersen, Hannah Nørtoft Frankel, Sandra Søgaard Tøttenborg, Karin Sørig Hougaard, Camilla Sandal Sejbaek

**Affiliations:** 1 Department of Occupational and Environmental Medicine, Copenhagen University Hospital – Bispebjerg and Frederiksberg, Copenhagen, Denmark.; 2 Department of Public Health, University of Copenhagen, Copenhagen, Denmark.; 3 National Institute of Occupational Health (STAMI), Oslo, Norway.; 4 National Research Centre for the Working Environment, Copenhagen, Denmark.; 5 Department of Public health, Section of Environmental Health, University of Copenhagen, Denmark.

**Keywords:** epidemiology, maternity leave, occupational medicine, sick leave

## Abstract

**Objectives:**

This study aimed to describe occupational, industrial, and temporal trends in relation to absence during pregnancy in the Danish workforce.

**Methods:**

The register-based national cohort DOC*X-Generation was used to identify all pregnancies among women (18–50 years) engaged in regular employment in Denmark 1998–2018. The cohort holds individual-level data on occupations coded according to the Danish versions of the International Standard Classification of Occupations and of EU’s nomenclature (NACE, revision 2). Data on absence from work was retrieved from the Danish Register for Evaluation and Marginalization. The study population comprised 884 616 pregnancies in 547 870 women.

**Results:**

In 48% of the included pregnancies, the women had at least one week with registered absence with a median of 8 weeks (5–95% percentile; 1–27 weeks). The highest frequencies of absence were observed among painters (75%) and women in the meat products manufacturing industry (68%), whereas the lowest were seen among professionals in physics, mathematics, engineering, and architecture (30%) and in the research and university education industry (32%). The difference between the lowest and highest number of cumulated weeks with absence was 9 weeks. From 1998–2018, the proportion of pregnancies with registered absence decreased, whereas the extent of absence per pregnancy increased.

**Conclusions:**

Absence during pregnancy was consistently high over time, but with vast differences across occupations and industries. A deeper understanding of underlying reasons for pregnancy-related absence is essential to develop targeted strategies for reducing absence, such as providing better opportunities for adjustments of work task early in pregnancy or other tailored interventions.

Absence during pregnancy is common in Denmark and the other Nordic countries. A national survey, published in 2010, found that two-thirds of Danish women were absent from work during pregnancy, approximately half for >4 weeks, thereby exceeding the rate of absence among non-pregnant women of fertile age manyfold (1) A Norwegian study from 2014 and several smaller Scandinavian studies found similar results (2–7). In a later Danish report based on data from 2011–2017, absence during pregnancy remained consistently frequent (8) In addition to societal consequences, extended periods of absence during pregnancy constitutes a problem for employers and colleagues but especially for the pregnant woman herself. Concerns about the employer’s reaction and the extra burden on colleagues can have a negative impact on the pregnant women’s mental health. Prolonged absence can increase risk of isolation and challenge the return to work after maternity leave (9–11).

Previous studies have demonstrated associations between occupational exposures – such as standing, walking, heavy lifting, shiftwork, and high strain work – and absence during pregnancy (6, 12–17). Some studies have indicated an exposure–response pattern with the risk of absence during pregnancy increasing with the number of occupational exposures experienced. However, much of the existing evidence is based on self-reported work exposures and data from selected populations, often with underrepresentation of women with lower levels of education (13, 18). Furthermore, existing studies often lack detailed information regarding the pregnant women’s occupations and industries. Finally, the methods used to quantify absence have varied, limiting comparability over time (1, 8, 13). More insight into the magnitude of absence over a longer time span and in an unselected population of pregnant employees is therefore warranted especially for women working in smaller industries and rare occupations and of lower socioeconomic position, where higher levels of occupational strain might be expected (19).

Using of the Danish nationwide register-based occupational generation cohort DOC*X-Generation (20), we aimed to (i) describe absence during pregnancy, according to occupation and industry, among women engaged in regular employment in Denmark and (ii) further describe the development in such absence from 1998 until 2018.

## Methods

DOC*X-Generation is a national occupational generation cohort including all pregnancies among employees (aged 1–50 years) in Denmark from 1977 to 2018. The cohort has been described in detail elsewhere (20, 21). Briefly, the cohort is based on several Danish registers and holds individual-level data on occupations coded according to DISCO-88 (the Danish 1988 version of the International Standard Classification of Occupations) and industries classified according to Dansk Branchekode 2007 (DB07, the Danish version of the EU’s nomenclature (NACE, revision 2). Pregnancies were identified and retrieved from the Danish Medical Birth Registry (DMBR; births), the Danish National Patient Register (DNPR; spontaneous abortions), and the National Abortion Register (induced abortions). Time of conception was calculated from registered gestational age based on midwives’ or doctors’ clinical assessments, date of last menstrual period or ultrasound estimation. If gestational age was missing, the median value of the duration of gestation on non-missing values for the specific outcome was assigned. In the present study, the underlying timescale was gestational weeks (GW), counted from the time of conception.

We used information on absence and maternity leave from the Danish Register for Evaluation and Marginalization (DREAM), a register under the Danish Agency for Labor Market and Recruitment (22). DREAM holds data on pregnancy-related benefits from 1998, thus the present study was based on register data from 1998 until and including 2018.

### Study population

Each individual pregnancy served as a unit in the cohort, meaning that each woman could contribute with more than one pregnancy. DOC*X-Generation has 1 659 823 pregnancies registered in the period 1998–2018. In Denmark, nearly all pregnant women attend their first pregnancy visit with their general physician around GW 6–10. Data from a Danish cohort showed that pregnant employees inform their employer about their pregnancy around GW 9–10 (23). Thus, pregnancies lasting <9 weeks were excluded (N=268 842). Furthermore, we excluded pregnancies among women: (i) with no registered job code in DOC*X-Generation for the year of conception (N=285 143); (ii) not registered in DREAM (N=513); (iii) registered with benefit transfers unrelated to employment (N=151 690); (iv) with supported employment (N=16 789); (v) registered in DREAM as living abroad (N=5 582); (vi) on sick or maternity leave during the week of conception (N=43 351); and (vii) <18 years of age (N=3 297). The final study population consisted of 884 616 pregnancies in 547 870 women (supplementary material, www.sjweh.fi/article/4245 figure S1).

### The Danish setting

According to Danish law, a woman is entitled to paid absence during pregnancy before maternity leave, if (i) a medical doctor finds that the pregnancy poses health risks to the woman or fetus if she continues working; or (ii) the nature of the work poses a risk to the fetus or the pregnancy, and the employer cannot offer suitable alternative work. In these situations, the employer is entitled to reimbursement (a financial compensation that employers can apply for from the municipality or state) from the first day of leave, and pregnancy-related absence will be registered in the DREAM register from day one of absence. If sickness is unrelated to the pregnancy, the absence is conceived as regular sick leave, with reimbursement starting after a so-called “period of waiting time”. The duration of these “periods of waiting time” has increased over time: 14 consecutive days (1989–2007), 21 consecutive days (2008–2012), and 30 consecutive days (from 2012 and onwards). The registration of sick leave in DREAM has changed accordingly. We therefore assigned all registered pregnancies a delay of reimbursement of 30 days to ensure harmonization across time periods.

In Denmark, all pregnant women are entitled to maternity leave 4 weeks prior to their due date (after GW 36+0). However, depending on collective agreement, many pregnant women can start maternity leave before this time. Hence, pregnant women in many public sector jobs (eg, healthcare personnel and school- and kindergarten teachers) may begin maternity leave after GW 32+0, pregnant women employed by the Danish State (eg, case worker) after GW 34+0, whereas pregnant women in private companies (eg, employee within service and craftsmanship) can start maternity leave after GW 36+0, with exceptions. Early maternity leave due to collective agreements is not registered in DREAM. To account for differences in duration of maternity leave before birth, frequencies of registered absence during pregnancy and cumulated absence by time period were described until GW 36+0 and GW 32+0, respectively.

### Industries and occupations

The DISCO-88 job classification system contains 372 different job codes at the most detailed (4-digit) level. To ensure adequate statistical power, we grouped job codes while mixing professional level and job characteristics to the smallest possible extent (supplementary table S1). Starting from the 4-digit level, the original job codes were kept as separate groups where possible (eg, occupational groups 21–25). As a next step, it was sought to combine job codes at the 4-digit level within the same 3-digit group (eg, occupational groups 04, 11, 12, and 20). If not possible, grouping of codes the 3-digit level within the same 2-digit group was pursued (eg, group 7). In some cases, job codes from different 1-digit groups were combined, if meaningful (eg, nurses and midwives in group 5). The grouping left some job codes with a limited number of pregnancies and somewhat related work tasks, these were grouped within the 1-digit level as “not classified” (NC). This grouping of jobs, based on the distribution of pregnancies and knowledge of the content of work, codes resulted in 38 occupational groups.

Industries were grouped according to the Danish Working Environment Authority´s (WEA) 37 groups, which is an aggregate of the 726 industry codes in DB07 (6-digit level) (24).

### Outcome

In the present study, absence during pregnancy was defined as absence consisting of both pregnancy-related absence (due to either abnormal course of pregnancy or presence of working conditions harmful to pregnancy) and sick-leave due to sickness unrelated to pregnancy. However, to illustrate the extent of both types of absence throughout pregnancy, these were also calculated separately and plotted as frequency of weekly absence in all pregnancies by type of absence

Data on absence during pregnancy was retrieved from the DREAM register, which includes all individuals who have received public benefit transfer in Denmark (including reimbursement to an employer). The register holds weekly information on type of benefit transfer with each type of having a unique code (eg, 890 sickness benefits and 881 maternity leave benefits). Maternity leave benefits and reimbursement in relation to pregnancy-related absence are registered with the same code (881). Registration in the register requires merely one day of absence during a week. Hence our outcome is “weeks *with* absence” with no specific indication of the numbers of days of absence. Only one code is registered weekly, and the codes are arranged hierarchically with the highest-ranking code being registered. Maternity leave and pregnancy-related absence benefit ranks higher than sickness benefit.

### Statistics

Absence during pregnancy until GW 36+0/32+0, was described stating absolute numbers and proportions for pregnancies in women with ≥1 week with a registered day of absence. Among these pregnancies, cumulated weeks with absence was calculated in total and according to occupational group, industrial group, and time-period (1998–2003, 2004–2008, 2009–2013, 2014–2018). Results were given as mean, median, and 5%-, 25%, 75% and 95% percentiles presented in boxplots.

Time-to-first absence during pregnancy, according to occupational and industrial groups, was illustrated by use of Kaplan Meier curves with pregnancy week as the underlying time scale. We followed the pregnant women from conception until their first absence, death, emigration, abortion, birth or start of GW 37 whichever came first. In addition, the risk of absence during pregnancy (until GW 36+0) among women in the different occupational and industrial groups was analyzed by Cox-regression and presented as hazard ratios (HR) with the largest occupational group and industrial group as references, respectively.

## Results

Of the included pregnancies, 11% terminated as abortions (both spontaneous and induced) and 4% resulted in preterm birth, defined as live births in GW 22–36 (table 1). The three occupational groups with the highest number of pregnancies consisted of administrative jobs [(i) clerks without customer contact, (ii) associate professionals in business and administration, and (iii) professionals at academic level (NC)], with >70 000 pregnancies in each group. They were followed by human-centered jobs within healthcare, education, personal care, and childcare (N ~ 28 000–54 000) and shop assistants (n ~38 000). The distribution of occupations reflected the sizes of the industrial groups with office work comprising the highest number of pregnancies (N=156 947) followed by residential care and home care services, hospitals, childcare services, and education (supplementary table S2a+b).

**Table 1:** Characteristics of the study population in relation to absence during pregnancy. N=884 616 pregnancies, 1998–2018. [GW=gestational week.]

Characteristics		Numbers of pregnancies (%)			Frequency of registered absence ^a^ during pregnancy until GW36+0	
		N	% ^b^		N	% ^c^
Total population		884 616	100.0		426 125	48.2
Age of the women (years) at conception						
	18–25	128 841	14.6		68 442	53.1
	26–30	336 551	38.0		168 549	50.1
	31–35	294 275	33.3		137 246	46.6
	36–40	109 533	12.4		46 719	42.7
	41–50	15 416	17.4		5169	33.5
Parity						
	0	370 620	41.9		183 794	49.6
	1	293 680	33.2		161 927	55.1
	2	100 078	11.3		58 220	58.2
	3–11	19 736	2.2		12 069	61.2
	Missing ^d^	100 502	11.4		10 115	10.1
Duration of pregnancies (GW)						
	9–21	100 171	11.3		9941	9.9
	22–36	36 055	4.1		32 167	89.2
	37–40	362 599	41.0		206 487	56.9
	≥41	385 791	43.6		177 530	46.0
Time periods						
	1998–2003	281 593	31.8		141 807	50.4
	2004–2008	232 531	26.3		121 764	52.4
	2009–2013	202 846	22.9		91 035	44.9
	2014–2018	167 646	19.0		71 519	42.7
Industrial groups with highest proportion of pregnancies in women with registered absence during pregnancy						
	Manufacture of meat products	5124	0.6		3502	68.3
	Residential centers and home help	87 984	9.9		54 963	62.5
	Cleaning industry	17 988	2.0		10 859	60.4
	Building completion and finishing	5196	0.6		3090	59.5
Industrial groups with lowest proportion of pregnancies in women with registered absence during pregnancy						
	Manufacture of chemicals and pharmaceuticals	13 769	1.6		4 776	34.7
	Energy, mining, and quarrying	2469	0.3		846	34.3
	Publishing, broadcasting, and motion pictures	16 979	1.9		5466	32.2
	Research and university education	18 279	2.1		5 874	32.1
Occupational groups with highest proportion of pregnancies in women with registered absence during pregnancy						
	Painters	3042	0.3		2284	75.1
	Food and beverage production workers	8245	1.0		5523	67.0
	Assembly workers	4943	0.6		3231	65.4
	Home-based personal care workers	36 374	4.1		23 417	64.3
Occupational groups with lowest proportion of pregnancies in women with registered absence during pregnancy						
	Senior officials and corporate managers	8881	1.0		3145	35.4
	Professionals at academic level ^e^	70 932	8.0		23 675	33.4
	Teachers in higher, secondary, vocational, and special education	20 418	2.3		6543	32.0
	Professionals (physics, mathematics, engineering, architects)	21 949	2.5		6464	29.5

^a^ ≥ 1 week (at least one day within) of registered absence. ^b^ Proportions (%) relative to column. ^c^ Proportions (%) relative to rows. ^d^ In case of registered abortion there is no information on parity. ^e^ Not classified.

**Figure 1:**
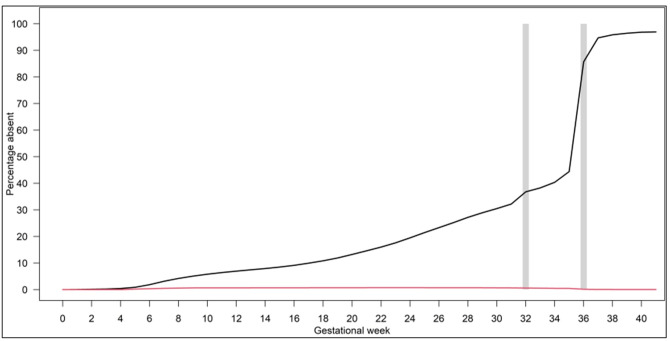
Frequency of weekly absence in all pregnancies by type of absence, N=884 616 pregnancies, 1998–2018. Red line: Sick leave unrelated to pregnancy; Black line: Pregnancy related absence. The vertical lines illustrate the time for maternity leave according to different collective agreements.

The analyses separated on absence during pregnancy showed an increasing frequency of weekly pregnancy-related absence throughout the pregnancy, whereas the frequency of weekly sick leave was steadily low (figure 1). In 48% of the included pregnancies, the women had ≥1 week with a registered day of absence during pregnancy (until GW 36+0) with a median of 8 weeks with absence (5–95% percentile; 1–27 weeks registered absence was observed most frequently in pregnancies among women aged 18–25 years (53%) and among those who had given birth before, with the proportion rising as the number of previous births increased (55–61%) (table 1).

Overall, pregnancy-related absence generally increased steadily throughout pregnancy, but with a sudden, large and very steep increase around GW 36, concomitant with the right to begin maternity leave 4 weeks before the due date according to Danish regulations. Smaller and less steep increases in pregnancy related absence were also seen around GW 32 and 34. The proportion of pregnancies in women with registered absence due to sick leave not related to pregnancy was very low and stable during pregnancy (figure 1).

### Time periods

The proportion of pregnancies with ≥1 week with registered absence until GW 36+0 appeared lower during the later time periods (2009–2013 and 2014–2018) with proportions of 45% and 43%, respectively, compared to the earlier time periods (1998–2003 and 2004–2008) with proportions of 50% and 52% (table 1). Conversely, among pregnancies with absence, the median number of cumulative weeks with registered absence increased, from 7 weeks in 1998–2003 to 9 weeks during the two latest periods (until GW 36+0). For cumulated weeks with absence until GW 32+0, the medians for each of the four time periods were 7, 8, 8, and 7 weeks of absence, respectively (supplementary figure S2a+b).

### Occupations

The highest proportion of pregnancies with ≥1 week with registered absence (until GW 36+0) was observed among painters (75%). Food and beverage production workers, assembly workers, and home-based personal care workers had the second highest rates of absence, all >64%. The lowest proportions (30–35%) were observed among (i) professionals (physics, mathematics, engineering, architects), (ii) teachers in higher, secondary, vocational, and special education, (iii) professionals at academic level (NC), and (iv) senior officials and corporate managers (table 1 and supplementary table S2a).

**Figure 2:**
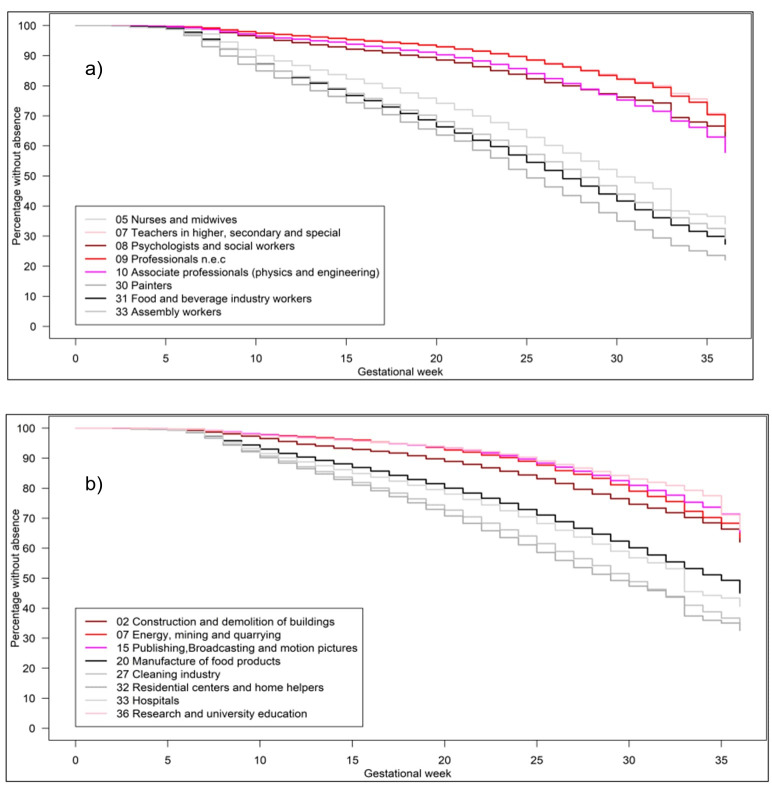
Time to first registered week with absence during pregnancy by occupational groups (a) or industrial groups (b), N=884 616 pregnancies, 1998–2018.

The steepest decline in the weekly proportion of pregnancies among women working during pregnancy was observed for painters, with 65% working around GW 20 decreasing to 25% around GW 36. Among food and beverage production workers, assembly workers, and home-based personal care workers a comparable, albeit less pronounced, pattern was observed. In the four mentioned occupations with the lowest proportion of pregnancies with absence, around 95% of the women were still working around GW 20 and 70% around GW 36 (figure 2a).

Painters had the highest increased risk (HR=2.77) of absence during pregnancy compared to the reference group (clerks without customer contact). In nine occupational groups, the risk of absence was lower than in the reference group (HR between 0.59 and 0.97). These nine occupational groups were all administrative and/or characterized by a high level of education. (Supplementary table 3a).

**Figure 3:**
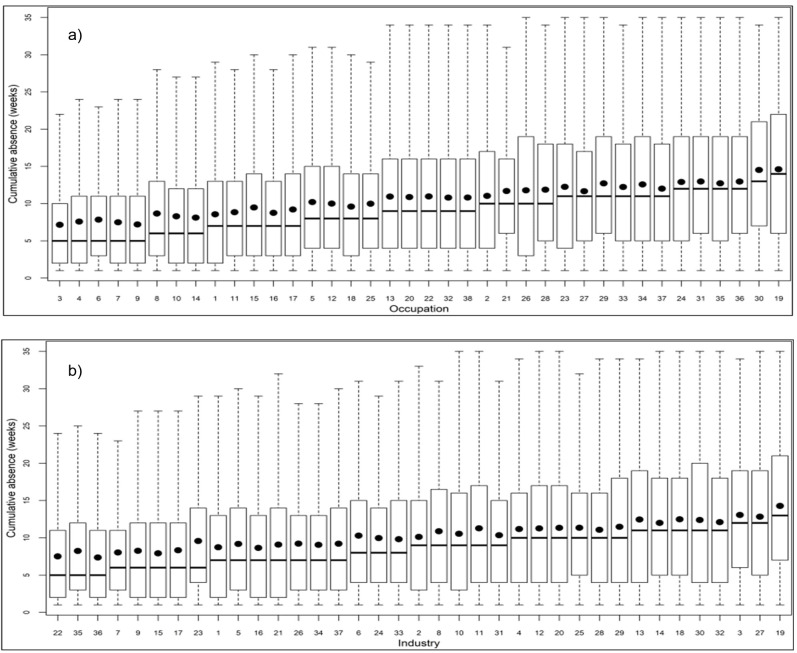
Cumulated weeks with absence by occupational group (a) or industrial group (b). Arranged by size of median cumulated weeks of absence, N=424 616 pregnancies, 1998–2019. NC=not classified. The thick horizontal line indicates the median, and the black dot represents the mean. The lower and upper bounds of the box represent the 25^th^ and 75^th^ percentiles, respectively. Whiskers denote the 5^th^ and 95^th^ percentiles. **A.** 1 Senior officials and corporate managers, 2 Managers (<10 employees), 3 Professionals (physics, mathematics, engineering, architects), 4 Medical doctors, dentists, and veterinarians, 5 Nurses and midwifes, 6 Primary School teachers, 7 Teachers in higher, secondary, vocational, and special education, 8 Psychologists and social workers, 9 Professionals at academic level NC, 10 Associate professionals (Physics and engineering), 11 Health associate professionals, 12 Teaching associate professionals (nursery and kindergarten), 13 Associate professionals (business and administration), 15 Associate professionals NC, 16 Clerks without customer contacts, 17 Customer service clerks, 18 Clerks NC, 19 Travel attendants and related workers, 20 Cooks and housekeepers, 21 Waiting staff and bartenders, 22 Childcare workers in private homes, 23 Institution-based personal care workers, 24 Home-based personal care workers, 25 Hairdressers, beauticians, and related workers, 26 Public safety workers, 27 Shop assistance, 28 Sales and service workers NC, 29 Skilled and unskilled agricultural, forest and fishery workers, 30 Painters, 31 Food and beverage production workers, 32 Skilled workers NC, 33 Assembly workers, 34 Production and plant operators, 35 Cleaners, janitors, and kitchen helpers, 36 Drivers and manual workers in construction, or manufacturing industries, 37 Unskilled workers NC. **B.** 1 Civil engineering, 2 Construction and demolition of buildings, 3 Building completion and finishing, 4 Retail trade, 5 Wholesale trade, 6 Manufacture of electronic components, 7 Energy, mining, and quarrying, 8 Repair and installation of machines, 9 Manufacture of chemicals and pharmaceuticals, 10 Manufacture of metals and machinery, 11 Manufacture of plastic, glass, and concrete, 12 Textile and paper products, 13 Manufacture and repair of vehicles, 14 Wood products and furniture, 15 Publishing, Broadcasting, and Motion pictures, 16 IT and telecommunications, 17 Administration, brokers, consulting, 18 Agriculture, forestry, and fishing, 19 Manufacture of meat products, 20 Manufacture of food products, 21 Defense, security, and justice act, 22 Religious institutions and funerals, 23 Water supply, sewage, and waste management, 24 Hairdressing and other personal service, 25 Hotel and other accommodation facilities, 26 Culture og sports, 27 Cleaning industry, 28 Restaurants and bars, 29 Transport of goods, 30 Transport of passengers, 31 Daycare (all ages), 32 Residential centers and home help, 33 Hospitals, 34 Health practitioners and veterinarians, 35 university education and training, 36 Research and university education, 37 Unstated.

In pregnancies with registered absence, the median number of cumulative weeks with absence overall increased as the duration of education decreased. The highest medians were seen among travel attendants (14 weeks), followed by painters (13 weeks). The lowest medians (5 weeks) were seen among teachers, professionals, medical doctors, dentists, and veterinarians (figure 3a).

### Industries

When absence was categorized by industrial group, the highest proportions of absence were observed in the manufacture of meat products group (68%) followed by residential institutions and home help (63%), cleaning, and building completing and finishing (both 60%). The lowest proportions were seen in the research and university education group (32%) (table 1 and supplementary table S2b).

Among the mentioned industrial groups, the steepest decline in the weekly proportion of pregnancies in women working during pregnancy was observed in the manufacture of meat products group with about 60% working around GW 20 decreasing to 30% around GW 36. Among the four industrial groups with the lowest proportion of pregnancies with absence [(i) research and university education, (ii) publishing, broadcasting, and motion pictures, (iii) energy, mining, and quarrying, and (iv) manufacture of chemicals and pharmaceuticals], the proportion of pregnancies in women still working was high and relatively stable during pregnancy with 95% working around GW 20 and 75% around GW 36 (figure 3b).

The highest risk of absence during pregnancy at any time was seen in the manufacture of meat group (HR 3.14) compared to the reference group (pregnancies among women working in the administration, brokers, or consulting industry). In eight industrial groups, the risk was lower compared to the reference group, most pronounced in the research and university education group (HR=0.80) (supplementary table S3b).

The highest median cumulative weeks with absence was 13 weeks, which was observed in the meat products manufacturing group, followed by the cleaning and building completing and finishing groups (12 weeks). The lowest median cumulated absence (5 weeks) was seen in the industrial groups of religious institutions and funerals and research and university education (figure 3b).

## Discussion

In this nationwide register-based study of pregnant women, almost half of the included pregnancies among women engaged in regular employment in Denmark 1998–2018 had registered absence from work. The median number of weeks with absence until GW 36+0 was 8 weeks, maternity leave excluded. Absence was most common among women aged 18–25 years and among parous women. Overall, we saw large differences between occupational and industrial groups with manual versus administrative work. This was obvious, both in relation to the proportions of pregnancies registered with absence (up to 45 percentage point difference) and the number of weeks with registered absence (up to 9 weeks difference). Overall, absence during pregnancy remained consistently high and largely unchanged over time.

A previous national analysis of pregnancy-related absence in Denmark, covering 2004–2007, reported that 2/3 of all pregnant women had absence during pregnancy with a mean absence of 48 days (1). We found a lower proportion of pregnancies with absence, which may be due to our exclusion of pregnancies among women with supported employment or changes in registration practices across time. In contrast, the median of 8 weeks with registered absence until GW 36+0, equaling 40 days (assuming absence all five workdays per week), corresponds roughly to the Danish analysis and is also consistent with findings from a national Norwegian study (7). Several studies from Europe, primarily of Scandinavian origin, describe prevalences of women with absence during pregnancy ranging from around 32% to 75%, based on self-reported outcomes (5, 15–17, 25, 26) Some studies also report cumulated days of absence with averages of 9.1 days (until GW 28) (16), 20 days (until GW 32) (5), and a median of 8 weeks of absence (until GW 32) (25).

In line with our findings, previous studies and national analyses have found the highest absence during pregnancy in the youngest age-groups and among parous women (1, 5, 7, 8, 16, 17, 25, 27). Also, in agreement with our findings, the Danish analysis covering 2004–2007 found the highest cumulative absence among industries with manual labor (eg, construction, farming, and food industry). Further, as in the present study, medium high absence was observed in industries within health- and social care (1).

Increase in absence as pregnancy progresses has also been described in several studies (2, 8, 28, 29). In our study, we further observed that the proportion of pregnant women still working decreased earlier in pregnancy and with higher proportions in occupational and industrial groups characterized by manual work than in those with administrative work. Many studies have shown associations between occupational strain, such as standing, walking, heavy lifting, shiftwork, high demands or low control, and absence during pregnancy (6, 12, 15–17, 30). In addition, studies have reported associations between both physical workload and shiftwork and adverse pregnancy outcomes (31–35), which is reflected in many national occupational health guidelines for pregnant workers (36–39). The higher prevalence of these factors in manual jobs might explain the present finding of the highest absence in manual occupations and the high absence in occupations within health- and social care, which often include manual person handlings, standing, walking, and shiftwork.

Even though absence during pregnancy is higher among manual workers, it is also relatively high in non-manual work. However, multiple factors unrelated to work are also associated with absence during pregnancy, eg, age, educational level, and general health (5, 16, 19, 25). This might explain why workplace interventions have not been able to prove any effect on absence nor on well-being among pregnant employees (28, 29, 40–42). Adjustment of work task and time schedules is nevertheless important to prevent negative pregnancy outcomes and pregnancy-related absence but does not occur to a sufficient extent (15, 43, 44), perhaps because the employer lacks knowledge or has many other considerations (45).

In the Nordic countries labor-market policies aim to accommodate work-organization- and tasks to ensure an appropriate and safe work environment for pregnant women to promote gender equality and counteract risk of marginalization because of weakened contact with workplaces and colleagues. A remarkable finding of this study is that absence during pregnancy has been stable at a high level during the long period of follow-up despite these incentives. Considering the meager effects of intervention in studies with primary focus on the workplace, it seems that a broader approach including also primary health and social care may be needed. Due to the importance of societal context our findings may have limited application outside comparable countries (19).

### Strengths and limitations

The present study is based on registry data, which, in contrast to most pregnancy cohorts, removes the risk of inclusion bias by self-selection and enables study of absence among nearly all pregnant women in all types of occupations and industries. Furthermore, the use of the DREAM database removes risk of recall bias, which is a known risk in studies based on self-reported absence (46–49) However, use of registry data relies on valid and consistent registration, which might not always be the case. In DOC*X, employees at workplaces with <10 employees will not necessarily have a registered DISCO-code because it is not mandatory and, therefore, results may not be fully generalizable to pregnant employees in small companies. The DREAM register contains data on all Danish citizens receiving social benefit transfers, based on registrations from public authorities and institutions, with absence-related data relying on employer reports. We believe that employer reporting is less of a concern, as pregnancy-related absence qualifies for reimbursement from day one, giving employers a financial incentive of reporting. Short-term sick leave unrelated to pregnancy lasting ≤30 consecutive days is not registered in DREAM. Although we have underestimated the true extent of total absence during pregnancy, we believe this is modest as non-pregnancy related sick leave was very low throughout the pregnancy weeks and because pregnancy related leave seldom is of short duration. For example, in a study on healthcare workers based on payroll data pregnancy-related absence was very seldom of shorter duration than one week (14). Hence, missing or unregistered sick leave does probably not explain the observed differences in absence during pregnancy between occupational and industrial groups. Conversely, the described absence during pregnancy could also be overestimated since weekly registration in DREAM required merely one day of absence. This rather crude registration implies that our findings are not fully comparable with assessments made of days of absence. As a conservative approach, we excluded pregnant women who were on sick or maternity leave at the time of conception to ensure all included individuals were exposed to work and at risk of work-related absence throughout the entire pregnancy. This was essential to maintain the validity of the time-to-absence analyses.

Throughout the pregnancy weeks, we observed that the proportions of pregnant women with absence increased. There was a steep increase in absence around GW 36, reflecting the possibility of maternity leave 4 weeks prior to the due date. However, absence also increased around GW 32 and 34 presumably representing the different collective and local agreements regarding the possibility for earlier maternity leave but overall, the ranking of occupational/industrial groups was the same with no sign of a systematic pattern of potential over- or underestimation of absence until GW 36+0 when comparing with the results describing absence until GW 32+0. Our primary results (until GW 36+0) include all absence but are probably less valid especially when comparing between different occupational or industrial groups. Also, when comparing time periods, changes in collective agreements over time can probably explain some of the difference in absence until GW36+0.

Another limitation is the change in duration of “periods of waiting time” in relation to sickness absence. Although we accounted for this by aligning all “periods of waiting time” to 30 days, description of changes over time can be challenging. Nevertheless, general sickness absence was very low and stable during pregnancy, and thus, of less concern in relation to absence during pregnancy.

### Concluding remarks

Absence during pregnancy was consistently high over time, but the extent differed vastly across occupations and industries. However, also in occupations and industries with assumed optimal working conditions, there is still a considerable amount of absence. A deeper understanding of the underlying reasons for pregnancy-related absence is essential to develop targeted strategies for reduction of absence, such as providing better opportunities for adjustments of work tasks early in pregnancy or other tailored interventions at the societal, industry or workplace level.

## Supplementary material

Supplementary material

## Declarations

During the preparation of this work, the author used ChatGPT to improve readability and language of the paper. After using this tool/service, the author(s) reviewed and edited the content as needed and takes full responsibility for the published article.

## Protection of research participants

The study was based on register data, and no approval from the Danish National Committee on Health Research was required. Permissions was obtained by the Regional Data Protection Authority (P-2019-217) and the study was approved by Statistics Denmark and the Danish Health and Medicines Authority.
