# Supplementary material for: Absence during pregnancy in the Danish workforce: occupational, industrial, and temporal trends in a nationwide register-based cohort study
Source: Scand J Work Environ Health. 2025 Oct 30;51(6):483–94. doi: 10.5271/sjweh.4245 (PMC12590490; doi:10.5271/sjweh.4245)
Supplement: Supplementary material [file SJWEH-51-483-S001.pdf]

Absence during pregnancy in the Danish workforce: occupational, industrial, and temporal trends in a nationwide register-based cohort study[1]

by Luise Mølenberg Begtrup, PhD,[2] *Esben Meulengracht Flachs, PhD, Regitze Sølling Wils, PhD, Ingrid Sivesind Mehlum, PhD, Jens Peter Ellekilde Bonde, DSc, Astrid Juhl Andersen, PhD, Hannah Nørtoft Frankel, MD, Sandra Søgaard Tøttenborg, PhD, Karin Sørig Hougaard, PhD, Camilla Sandal Sejbaek, PhD*

1. Supplementary material
2. Correspondence to: Luise Mølenberg Begtrup, Department of Occupational and Environmental Medicine, Copenhagen University Hospital – Bispebjerg and Frederiksberg, Copenhagen, Denmark. [E-mail: Luise.moelenberg.begtrup.02@regionh.dk]

Supplementary figure S1. Flowchart, study population DOC*X-Generation

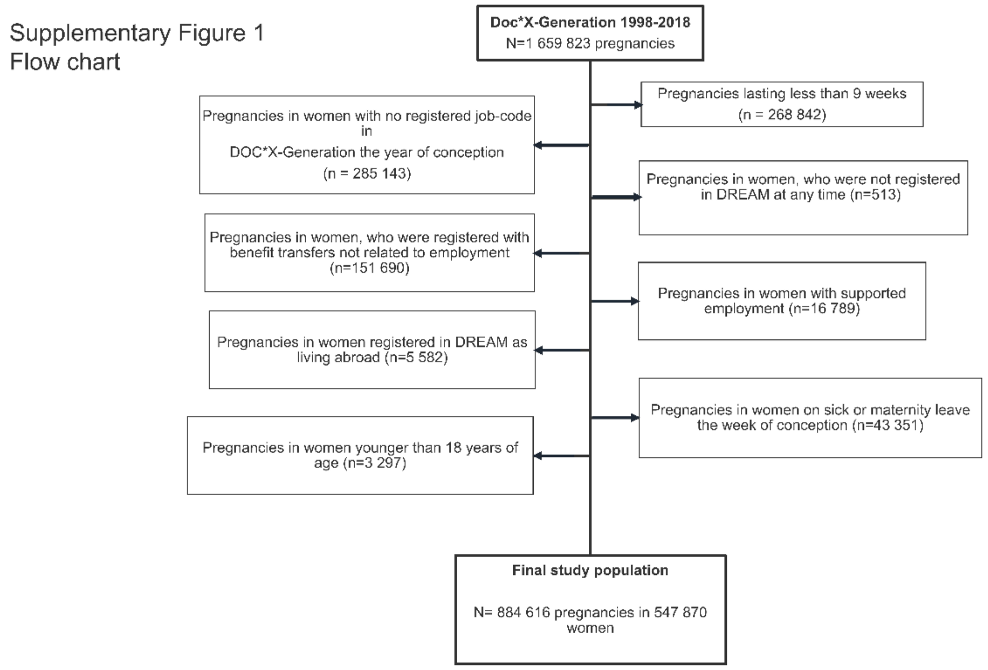

Supplementary Table S1. Description of the 38 occupational groups in relation to ISCO/ DISCO-88 codes and names in English and Danish

Occupational Groups	ISCO/DISCO-88 codes	English names (ISCO)	Danish names (DISCO)
01 Senior officials and corporate managers	11	Legislators and senior officials	Lovgivningsarbejde samt ledelse I offentlig administration og interesseorganisationer
	12	Directors and chief executives	Ledelse i virksomheder med 10 eller flere beskæftigede
02 Managers (< 10 employees)	13	Managers of small enterprises	Ledelse i virksomheder med færre end 10 beskæftigede
03 Professionals (physics, mathematics, engineering, architects)	21	Physical, mathematical, and engineering science professionals	Forskning og/eller anvendelse af færdigheder inden for de ikke-biologiske grene af naturvidenskab samt datalogi, statistik, arkitektur og tekniske videnskaber
04 Medical doctors, dentists, and veterinarians	2221	Medical doctors	Lægearbejde
	2222	Dentists	Tandlægearbejde
	2223	Veterinarians	Veterinærarbejde
05 Nurses and midwifes	2230	Nursing and midwifery professionals	Jordemoderarbejde, overordnet sygeplejearbejde mv.
	323	Nursing and midwifery associate professionals	Sygeplejearbejde
06 Primary School teachers	233	Primary and pre-primary education teaching professionals	Undervisning i folkeskoler og lignende
07 Teachers in higher, secondary, vocational, and special education	231	College, university, and higher education teaching professionals	Undervisning på universiteter og højere læreanstalter
	232	Secondary education teaching professionals	Undervisning på gymnasier, erhvervsskoler med videre
	234	Special education teaching professionals	Undervisning af handicappede mennesker
08 Psychologists and social workers	2445	Psychologists	Arbejde med emner indenfor psykologi
	2446	Social work professionals	Overordnet socialrådgivningsarbejde
	3460	Social work associate professionals	Socialt vejlednings- og omsorgsarbejde
09 Professionals at academic level n.e.c.	221	Life science professionals	Arbejde med emner indenfor de biologiske grene af naturvidenskab
	235	Other teaching professionals	Arbejde vedrørende undervisning i
	241	Business professionals	øvrigt
			Arbejde med emner vedrørende
	242	Legal professionals	virksomheders organisation og
	243	Archivists, librarians, and related information professionals	økonomi<br>Juridisk præget arbejde
	2441	Economists	Kulturformidling og informationsarbejde
	2442	Sociologists, anthropologists, and related professionals	Arbejde med emner indenfor
	2443	Philosophers, historians, and political scientists	samfundsøkonomi<br>Arbejde med emner indenfor sociologi
	2444	Philologists, translators and interpreters	og antropologi<br>Arbejde med emner indenfor filosofi og
	245	Writers and creative or performing artists	historie<br>Arbejde med emner indenfor
	246	Religious professionals	sprogvidenskab
	247		Journalist-, kunst og skribentarbejde

		Public service administrative professionals	Arbejde indenfor religion<br>Arbejde med administration af lovgivningen inden for den offentlige sektor
10 Associate professionals (Physics and engineering)	31	Physical and engineering science associate professionals	Teknikerarbejde inden for ikke-biologiske emner
11 Health associate professionals	3224	Optometrists and opticians	Optikerarbejde
	3225	Dental assistants	Assistentarbejde vedrørende tandpleje
	3226	Physiotherapists and related associate professionals	Arbejde med emner inden for fysioterapi, kiropraktik mv
	3227	Veterinary assistants	Assistentarbejde hos dyrlæge
	3229	Health associate professionals (except nursing) not elsewhere classified	Arbejde med emner indenfor ergoterapi, zoneterapi, yoga mv.
12 Teaching associate professionals (nursery and kindergarten)	331	Primary education teaching associate professional	Skoleundervisning af børn under den undervisningspligtige alder
	332	Pre-primary education teaching associate professionals	Pædagogisk arbejde med børn under den undervisningspligtige alder
13 Teaching associate professionals (special education and care)	333	Special education teaching associate professionals	Omsorgsarbejde med handicappede mennesker
14 Associate professionals (business and administration)	34	Other associate professionals	Arbejde med salg, finansiering, forretningsservice, administration mv
15 Associate professionals n.e.c.	321	Life science technicians and related associate professional	Teknikerarbejde inden for biologi, medicin, landbrug mv.
	3221	Medical assistants	Assistentarbejde og rådgivning vedr. sundhed, fødsel mv
	3222	Hygienists, health and environmental officers	Assistentarbejde og rådgivning vedr. hygiejne
	3223	Dieticians and nutritionists	Assistentarbejde og rådgivning vedr. kostforplejning på hospitaler og lign. institutioner
	3228	Pharmaceutical assistants	Assistentarbejde inden for farmaci
	334	Other teaching associate professionals	Undervisnings- og omsorgsarbejde i øvrigt
16 Clerks without customer contacts	41	Office clerks	Internt kontorarbejde
17 Customer service clerks	42	Customer services clerks	Kontorarbejde med kundebetjening
18 Clerks n.e.c.	4	If ISCO code with only one ciffer	If DISCO code with only one ciffer
19 Travel attendants and related workers	511	Travel attendants and related workers	Servicearbejde I forbindelse med transport og rejser
20 Cooks and housekeepers	5121<br>5122	Housekeepers and related workers<br>Cooks	Generelt husholdningsarbejde<br>Tilberedning af måltider
21 Waiting staff and bartenders	5123	Waiters, waitresses, and bartenders	Serveringsarbejde
22 Childcare workers in private homes	5131	Child-care workers	Børnepasning I private hjem
23 Institution-based personal care workers	5132	Institution-based personal care workers	Plejearbejde på institutioner
24 Home-based personal care workers	5133	Home-based personal care workers	Omsorgsarbejde i private hjem
25 Hairdressers, beauticians, and related workers	5141	Hairdressers, barbers, beauticians, and related workers	Personpleje
26 Public safety workers	345	Police inspectors and detectives	Politimæssigt undersøgelsesarbejde
	516	Protective services workers	Overvågnings- og redningsarbejde

27 Shop assistance	522	Shop, stall and market salespersons and demonstrators	Ekspedient-, kasse og demonstrationsarbejde
28 Sales and service workers n.e.c.	5139	Personal care and related workers not elsewhere classified	Omsorgs- og plejearbejde i øvrigt
	5142	Companions and valets	Kammertjener arbejde
	5143	Undertakers and embalmers	Bedemandsarbejde
	5149	Other personal services workers not elsewhere classified	Servicearbejde for privatpersoner i øvrigt
	521	Fashion and other models	Modelarbejde
29 Skilled and unskilled agricultural, forest and fishery workers	61	Skilled agricultural and fishery workers	Arbejde inden for landbrug, gartneri, skovbrug, jagt og fiskeri, der forudsætter færdigheder på grundniveau
	92	Agricultural, fishery and related labourers	Medhjælp inden for landbrug, gartneri, fiskeri og skovbrug
30 Painters	7141	Painters and related workers	Maler- og tapetsererarbejde, herunder skibsmalerog skiltemalerarbejde
	7142	Not in ISCO	Sprøjtelakeringsarbejde
31 Food and beverage production workers	741	Food processing and related trades workers	Arbejde inden for nærings- og nydelsesindustrien
	827	Food and related products machine operators	Betjening af maskiner indenfor nærings- og nydelsesindustrien
32 Skilled workers n.e.c.	711	Miners, shotfirers, stone cutters and carvers	Mine- og stenhuggerarbejde mv
	712	Building frame and related trades workers	Bygningsarbejde (basis)
	713	Building finishers and related trades workers	Bygningsarbejde (finish)
	7143	Building structure cleaners	Arbejde med bygningsrengøring (skorstensfejning)
	72	Metal, machinery, and related trades workers	Metal- og maskinarbejde
	73	Precision, handicraft, craft printing and related trades workers	Præcisionshåndværk, grafisk arbejde og lignende
	743	Textile, garment, and related trades workers	Tekstil og beklædningsarbejde
	744	Pelt, leather, and shoemaking trades workers	Skind-, læder- og skotøjsarbejde
33 Assembly workers	828	Assemblers	Monterings- og samlebåndsarbejde
34 Production and plant operators	81	Stationary plant and related operators	Arbejde med stationære procesanlæg
	821	Metal- and mineral-products machine operators	Betjening af maskiner inden for metal- og mineralindustrien
	822	Chemical-products machine operators	Betjening af maskiner inden for den kemiske industri, eksklusive procesanlægsarbejde
	823	Rubber- and plastic-products machine operators	Betjening af maskiner inden for gummi- og plastindustrien
	824	Wood-products machine operators	Betjening af maskiner inden for træindustrien
	825		Betjening af maskiner inden for tekstil-, skind- og lædervareindustrien
	829	Printing-, binding- and paper-products machine operators	Betjening af industrimaskiner i øvrigt

		Other machine operators not elsewhere classified	
35 Cleaners, janitors, and kitchen helpers	913	Domestic and related helpers, cleaners, and launderers	Rengørings- og køkkenhjælpsarbejde
	914	Building caretakers, window and related cleaners	Arbejde med bygningsrengøring og vinduespolering
36 Drivers and manual workers in construction, or manufacturing industries	83<br>93	Drivers and mobile plant operators<br>Labourers in mining, construction, manufacturing, and transport	Transport og anlægsarbejde<br>Manuelt arbejde indenfor bygge- og anlægssektoren, transportsektoren samt fremstillingsvirksomhed
37 Unskilled workers n.e.c.	911	Street vendors and related workers	Salgs- og servicearbejde primært ved telefon
	912	Shoe cleaning and other street services elementary occupations	Arbejde med skopudsning og lignende former for gadeservice
	915	Messengers, porters, doorkeepers, and related workers	Budtjeneste, vagtarbejde samt arbejde med måleaflæsning mv.
	916	Garbage collectors and related labourers	Renovations-, gadefejningsarbejde og lignende, eksklusive kørsel med renovationsvogne

ISCO: International Standard Classification of occupations version 88 , DISCO: The Danish version of the International standard classification of occupations version 88

nec: not else classified

Supplementary table S2a. Characteristics of the **occupational groups** according to prevalence of pregnancies in mothers with standard employment and absence during pregnancy in DOC*X-Generation from 1998-2018. Ranked by size of proportion of absence until gestational week (GW) 36+0

	Total		Registered absence during pregnancy (until **GW 32+0**)		Registered absence during pregnancy (until **GW 36+0**)	
	(N=884 616)		(N = 328 636)		(N = 426 125))	
**Occupational groups**	N	%[a]	N	%[b]	N	%[b]
30 Painters	3042	0.3	2121	69.7	2284	75.1
31 Food and beverage production workers	8245	0.9	4928	59.8	5523	67.0
33 Assembly workers	4943	0.6	2844	57.5	3231	65.4
24 Home-based personal care workers	36 374	4.1	20 768	57.1	23 417	64.4
23 Institution-based personal care workers	27 664	3.1	15 251	55.1	17 698	64.0
19 Travel attendants and related workers	2594	0.3	1438	55.4	1641	63.3
34 Production and plant operators	7471	0.8	4089	54.7	4673	62.5
05 Nurses and midwives	54 485	6.2	28 196	51.8	33 802	62.0
26 Public safety workers	3054	0.4	1513	49.5	1882	61.6
29 Skilled and unskilled agricultural, forest and fishery workers	7465	0.8	3905	52.3	4510	60.4
35 Cleaners, janitors, and kitchen helpers	26 288	3.0	13 658	52.0	15 828	60.2
36 Drivers and manual workers in construction or manufacturing	8897	1.0	4628	52.0	5236	58.9
13 Teaching associate professionals (special education and care)	15 873	1.8	7488	47.2	9296	58.6
12 Teaching associate professionals (nursery and kindergarten)	47 609	5.4	21 729	45.6	27 707	58.2
25 Hairdressers, beauticians, and related workers	10 474	1.2	5005	47.8	6068	58.1
20 Cooks and house keepers	12 502	1.4	5698	45.6	7068	56.5
27 Shop assistance	38 339	4.3	18 195	47.5	21 311	55.6
22 Childcare workers in private homes	27 896	3.2	12494	44.8	15 347	55.0
21 Waiting staff and bartenders	2789	0.3	1306	46.8	1517	54.4
11 Health associate professionals	25 366	2.9	9488	37.4	12 646	49.9
06 Primary school teachers	41 376	4.7	13 334	32.2	20 088	48.5
28 Sales and service workers n.e.c.	2914	0.3	1178	40.2	1413	48.5
15 Associate professionals n.e.c.	19 817	2.2	6873	34.7	9576	48.3
37 Unskilled workers n.e.c	2999	0.3	1190	39.7	1445	48.2
32 Skilled workers n.e.c.	7737	0.9	2865	37.1	3595	46.5
02 Managers (< 10 employees)	4471	0.5	1615	36.1	2008	44.9
18 Clerks n.e.c.	26 005	2.9	8537	32.8	11 307	43.5
16 Clerks without customer contacts	76 064	8.6	23 139	30.4	33 003	43.3
17 Customer service clerks	24 593	2.8	7942	32.2	10 602	43.1
38 Unstated	66 857	7.6	22 213	33.2	28 441	42.5
10 Associate professionals (physics and engineering)	16 721	1.9	4564	27.3	6584	39.4
04 Medical doctors, dentists, and veterinarians	15 780	1.8	4280	27.1	6148	39.0
14 Associate professionals (business and administration)	74 358	8.4	18 420	24.8	27275	36.7
08 Psychologists and social workers	11 374	1.3	2982	26.2	4110	36.1
01 Senior officials and corporate managers	8881	1.0	2198	24.7	3145	35.4
09 Professionals at academic level n.e.c	70 932	8.0	14 478	21.4	23 675	33.4
07 Teachers in higher, secondary, vocational, and special education	20 418	2.3	3998	19.6	6543	32.0
03 Professionals (physics, mathematics, engineering, architects)	21 949	2.5	4088	18.6	6464	29.5

GW Gestational week
nec not else classified

a Proportions (%) relative to coloum

b Proportions (%) relative to rows (according to GW 33 and GW 37)

Supplementary table S2b. Characteristics of the **industrial groups** according to prevalence of pregnancies in mothers with standard employment and absence during pregnancy in DOC*X-Generation from 1998-2018. Ranked by size of proportion of absence until gestational week (GW) 36+0

	Total (N=884 616)		Registered absence during pregnancy (until **GW 32+0**) (N = 328 636)		Registered absence during pregnancy (until **GW 36+0**) (N = 426 125)	
**Industrial groups**	N	%[a]	N	%[b]	N	%[b]
19 Manufacture of meat products	5124	0.6	3219	62.8	3502	68.3
32 Residential centers and home help	87 984	9.9	46 674	53.0	54 963	62.5
27 Cleaning industry	17 988	2.0	9411	52.3	10 859	60.4
03 Building completion and finishing	5196	0.6	2711	52.2	3090	59.5
18 Agriculture, forestry, and fishing	5838	0.7	2858	49.0	3373	57.8
31 Daycare (all ages)	80 620	9.1	36 038	44.7	45 538	56.5
24 Hairdressing and other personal service	11 625	1.3	5370	46.2	6556	56.4
33 Hospitals	87 711	9.9	39 639	45.2	49 368	56.3
30 Transport of passengers	5781	0.7	2572	44.5	3213	55.6
04 Retail trade	69 262	7.8	31 074	44.9	37 221	53.7
29 Transport of goods	11 758	1.3	5090	43.3	6275	53.4
14 Wood products and furniture	4994	0.6	2146	43.0	2604	52.1
21 Defense, security, and justice act	11 233	1.3	4014	35.7	5808	51.7
20 Manufacture of food products	10 826	1.2	4596	42.5	5543	51.2
25 Hotel and other accommodation facilities	6781	0.8	2851	42.0	3456	51.0
06 Manufacture of electronic components	9798	1.1	3709	37.9	4683	47.8
13 Manufacture and repair of vehicles	2101	0.2	816	38.8	993	47.3
28 Restaurants and bars	14 493	1.6	5570	38.4	6839	47.2
34 Health practitioners and veterinarians	29 024	3.3	10 221	35.2	13 708	47.2
12 Textile and paper products	5933	0.7	2246	37.9	2796	47.1
35 Non-university education and training	78 028	8.8	24 391	31.3	36 521	46.8
23 Water supply, sewage, and waste management	1176	0.1	380	32.3	548	46.6
11 Manufacture of plastic, glass, and concrete	7670	0.9	2794	36.4	3478	45.3
10 Manufacture of metals og machinery	13 587	1.5	4688	34.5	5914	43.5
08 Repair and installation of machines	853	0.1	292	34.2	366	42.9
22 Religious institutions and funerals	2623	0.3	564	21.5	1003	38.2
17 Administration, brokers, consulting	156 947	17.8	40 309	25.7	59 012	37.6
02 Construction and demolition of buildings	2121	0.2	618	29.1	796	37.5
05 Wholesale trade	40 931	4.6	11 004	26.9	15 094	36.9
26 Culture og sports	9925	1.1	2600	26.2	3612	36.4
16 IT and telecommunications	11 559	1.3	2923	25.3	4107	35.5
01 Civil engineering	1706	0.2	419	24.6	603	35.3
37 Unstated	21 924	2.5	5753	26.2	7721	35.2
09 Manufacture of chemicals and pharmaceuticals	13 769	1.6	3413	24.8	4776	34.7
07 Energy, mining, and quarrying	2469	0.3	582	23.6	846	34.3
15 Publishing, Broadcasting, and Motion pictures	16 979	1.9	3627	21.4	5466	32.2
36 Research and university education	18 279	2.1	3454	18.9	5874	32.1

GW Gestational week
nec not else classified
a Proportions (%) relative to coloum
b Proportions (%) relative to rows (according to GW 33 and GW 37)

Supplementary Figure S2a. Cumulated absence (until gestational week 36+0) by time period, N=884,616 pregnancies, 1998-2018

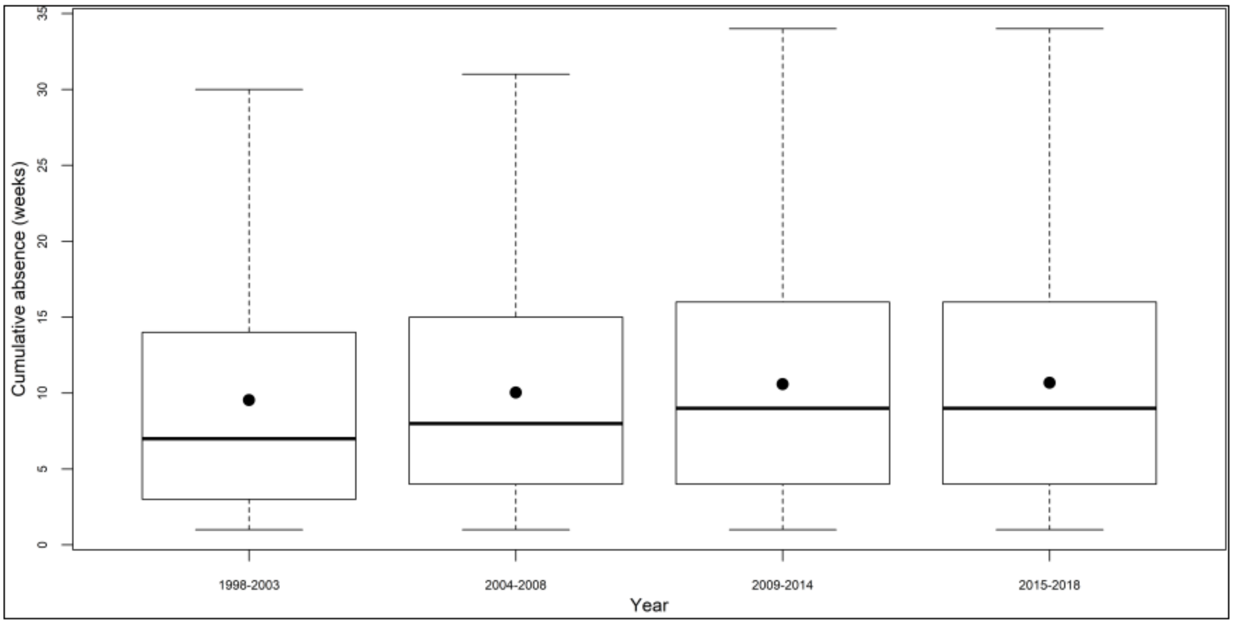

The thick horizontal line indicates the median, and the black dot represents the mean. The lower and upper bounds of the box represent the 25th and 75th percentiles, respectively. Whiskers denote the 5th and 95th percentiles.

Supplementary Figure S2b. Cumulated absence (until gestational week 32+0) by time period, N=884,616 pregnancies, 1998-2018

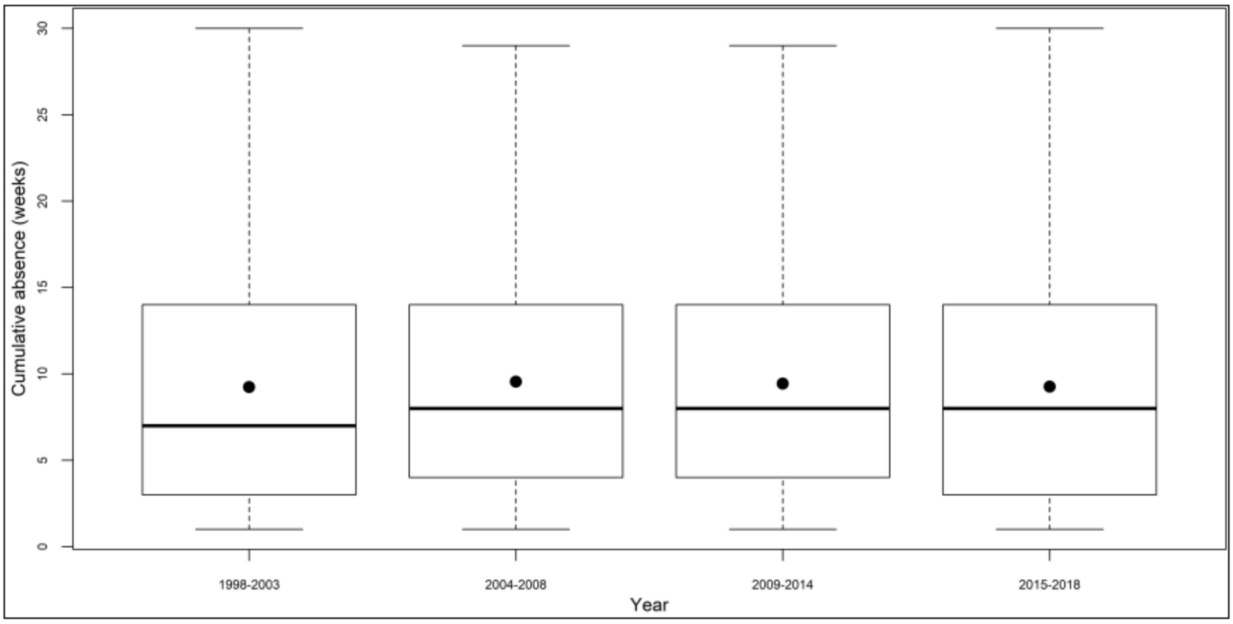

The thick horizontal line indicates the median, and the black dot represents the mean. The lower and upper bounds of the box represent the 25th and 75th percentiles, respectively. Whiskers denote the 5th and 95th percentiles.

Supplementary table S3a. Occupational group and risk of absence during pregnancy (Until gestational week 36+0) given as hazard ratios (HR) with the largest occupational group (Clerks without customer contact) as reference. The DOC*X-Generation, 1998-2018. N=888 616 pregnancies

**Occupational groups**	N	%[a]	HR	95% Confidence interval
30 Painters	3042	0.3	2.77	2.64-2.90
31 Food and beverage production workers	8245	0.9	2.32	2.25-2.39
24 Home-based personal care workers	36 374	4.1	2.21	2.18-2.25
33 Assembly workers	4943	0.6	2.18	2.10-2.26
19 Travel attendants and related workers	2594	0.3	2.17	2.06-2.28
23 Institution-based personal care workers	27 664	3.1	2.08	2.04-2.11
34 Production and plant operators	7471	0.8	2.03	1.98-2.10
35 Cleaners, janitors, and kitchen helpers	26 288	3.0	1.92	1.88-1.96
36 Drivers and manual workers in construction or manufacturing	8897	1.0	1.87	1.81-1.92
05 Nurses and midwives	54 485	6.2	1.85	1.82-1.88
26 Public safety workers	3054	0.4	1.84	1.32-1.40
29 Skilled and unskilled agricultural, forest and fishery workers	7465	0.8	1.78	1.72-1.84
21 Waiting staff and bartenders	2789	0.3	1.72	1.63-1.82
13 Teaching associate professionals (special education and care)	15 873	1.8	1.64	1.60-1.68
20 Cooks and house keepers	12 502	1.4	1.61	1.57-1.66
12 Teaching associate professionals (nursery and kindergarten)	47 609	5.4	1.60	1.58-1.63
22 Childcare workers in private homes	27 896	3.2	1.57	1.54-1.60
27 Shop assistance	38 339	4.3	1.57	1.54-1.59
25 Hairdressers, beauticians, and related workers	10 474	1.2	1.36	1.32-1.40
28 Sales and service workers n.e.c.	2914	0.3	1.32	1.25-1.39
37 Unskilled workers n.e.c	2999	0.3	1.27	1.20-1.34
11 Health associate professionals	25 366	2.9	1.17	1.14-1.19
15 Associate professionals n.e.c.	19 817	2.2	1.15	1.12-1.17
06 Primary school teachers	41 376	4.7	1.14	1.12-1.16
32 Skilled workers n.e.c.	7737	0.9	1.13	1.09-1.17
02 Managers (< 10 employees)	4471	0.5	1.08	1.03-1.13
**16 Clerks without customer contacts**	**76 064**	**8.6**	**1**	**Ref.**
17 Customer service clerks	24 593	2.8	1.00	0.98-1.03
38 Unstated	66 857	7.6	0.99	0.98-1.01
18 Clerks n.e.c.	26 005	2.9	0.97	0.95-0.99
10 Associate professionals (physics and engineering)	16 721	1.9	0.87	0.84-0.89
04 Medical doctors, dentists, and veterinarians	15 780	1.8	0.80	0.77-0.82
14 Associate professionals (business and administration)	74 358	8.4	0.78	0.77-0.79
01 Senior officials and corporate managers	8881	1.0	0.77	0.74-0.80
08 Psychologists and social workers	11 374	1.3	0.76	0.73-0.78
09 Professionals at academic level n.e.c	70 932	8.0	0.68	0.67-0.69
07 Teachers in higher, secondary, vocational, and special education	20 418	2.3	0.65	0.64-0.67
03 Professionals (physics, mathematics, engineering, architects)	21 949	2.5	0.59	0.57-0.61

nec not else classified

a Proportions (%) relative to column

Supplementary table S3b. Industrial groups and risk of absence during pregnancy (until gestational week 36+0) given as hazard ratios (HR) with the largest industry group (Administration, brokers, consulting) as reference. The DOC*X-Generation, 1998-2018. N=888 616 pregnancies

**Industrial groups**	N	%[a]	HR	95% Confidence interval
19 Manufacture of meat products	5124	0.6	3.14	3.04-3.26
32 Residential centers and home help	87 984	9.9	2.41	2.38-2.44
27 Cleaning industry	17 988	2.0	2.31	2.26-2.36
03 Building completion and finishing	5196	0.6	2.00	1.92-2.08
18 Agriculture, forestry, and fishing	5838	0.7	1.98	1.91-2.05
30 Transport of passengers	5781	0.7	1.93	1.86-2.00
31 Daycare (all ages)	80 620	9.1	1.92	1.90-1.94
33 Hospitals	87 711	9.9	1.88	1.85-1.90
29 Transport of goods	11 758	1.3	1.80	1.75-1.85
04 Retail trade	69 262	7.8	1.75	1.73-1.77
14 Wood products and furniture	4994	0.6	1.70	1.63-1.77
25 Hotel and other accommodation facilities	6781	0.8	1.69	1.60-1.70
20 Manufacture of food products	10 826	1.2	1.64	1.60-1.70
24 Hairdressing and other personal service	11 625	1.3	1.60	1.55-1.64
21 Defense, security, and justice act	11 233	1.3	1.58	1.53-1.62
28 Restaurants and bars	14 493	1.6	1.55	1.51-1.60
13 Manufacture and repair of vehicles	2101	0.2	1.47	1.38-1.57
06 Manufacture of electronic components	9798	1.1	1.46	1.41-1.50
12 Textile and paper products	5933	0.7	1.41	1.36-1.47
11 Manufacture of plastic, glass, and concrete	7670	0.9	1.38	1.33-1.43
23 Water supply, sewage, and waste management	1176	0.1	1.36	1.25-1.49
35 Non-university education and training	78 028	8.8	1.34	1.32-1.35
34 Health practitioners and veterinarians	29 024	3.3	1.30	1.27-1.32
10 Manufacture of metals and machinery	13 587	1.5	1.27	1.24-1.31
08 Repair and installation of machines	853	0.1	1.15	1.03-1.29
22 Religious institutions and funerals	2623	0.3	1.01	0.95-1.08
**17 Administration, brokers, consulting**	**156 947**	**17.8**	**1**	**Ref.**
26 Culture og sports	9925	1.1	1.00	0.96-1.03
05 Wholesale trade	40 931	4.6	0.98	0.96-0.99
02 Construction and demolition of buildings	2121	0.2	0.96	0.89-1.03
37 Unstated	21 924	2.5	0.96	0.93-0.98
16 IT and telecommunications	11 559	1.3	0.94	0.91-0.98
01 Civil engineering	1706	0.2	0.93	0.86- 1.02
09 Manufacture of chemicals and pharmaceuticals	13 769	1.6	0.92	0.89-0.95
07 Energy, mining, and quarrying	2469	0.3	0.88	0.82-0.95
15 Publishing, Broadcasting, and Motion pictures	16 979	1.9	0.83	0.80-0.85
36 Research and university education	18 279	2.1	0.80	0.77-0.82

nec not else classified

a Proportions (%) relative to column
